# sFlt-1/PlGF Ratio in Prediction of Short-Term Neonatal Outcome of Small for Gestational Age Neonates

**DOI:** 10.3390/children8080718

**Published:** 2021-08-23

**Authors:** Jacek Witwicki, Katarzyna Chaberek, Natalia Szymecka-Samaha, Adam Krysiak, Paweł Pietruski, Katarzyna Kosińska-Kaczyńska

**Affiliations:** 1 Department of Neonatology, Centre of Postgraduate Medical Education, Cegłowska 80, 01-809 Warsaw, Poland; jacekmichalw@gmail.com (J.W.); adam.krysiak@cmkp.edu.pl (A.K.); 2 2nd Department of Obstetrics and Gynecology, Center of Postgraduate Medical Education, Cegłowska 80, 01-809 Warsaw, Poland; chaberek.katarzyna@gmail.com (K.C.); szymecka.natalia@gmail.com (N.S.-S.); 3 Independent Public Complex of Healthcare Institutions in Pułtusk, 3 Maja 5, 06-100 Pułtusk, Poland; ppietruski@gmail.com

**Keywords:** fetal growth restriction, small for gestational age, neonatal outcome, prediction, PlGF, sFlt-1

## Abstract

Background: Small for gestational age is a pregnancy complication associated with a variety of adverse perinatal outcomes. The aim of the study was to investigate if sFlt-1/PlGF ratio is related to adverse short-term neonatal outcome in neonates small for gestational age in normotensive pregnancy. Methods: A prospective observational study was conducted. Serum sFlt-1/PlGF ratio was measured in women in singleton gestation diagnosed with fetus small for gestational age. Short-term neonatal outcome analyzed in the period between birth and discharge home. Results: Eighty-two women were included. Women with sFlt-1/PlGF ratio ≥33 gave birth to neonates with lower birthweight at lower gestational age. Neonates from high ratio group suffered from respiratory disorders and NEC significantly more often. They were hospitalized at NICU more often and were discharged home significantly later. sFlt-1/PlGF ratio predicted combined neonatal outcome with sensitivity of 73% and specificity of 82.2%. Conclusions: sFlt-1/PlGF ratio is a useful toll in prediction of short-term adverse neonatal outcome in SGA pregnancies.

## 1. Introduction

Small for gestational age (SGA) and fetal growth restriction (FGR) are complications of pregnancy that have been associated with a variety of adverse perinatal outcomes [1]. In FGR the fetus does not reach its biological and genetical growth potential, which may be because of a variety of factors [2]. SGA if commonly defined as fetal weigh below the 10th centile. As FGR definition was extremely heterogeneous across the world and in available research data, in 2016 it was determined by the expert consensus through a Delphi procedure [2]. According to the consensus early FGR is diagnosed before 32 weeks of gestation with three solitary parameters: abdominal circumference (AC) < 3rd centile, estimated fetal weight (EFW) < 3rd centile and absent end-diastolic flow in the umbilical artery (UA), or with four contributory parameters: AC or EFW < 10th centile combined with a pulsatility index (PI) > 95th centile in either the UA or uterine artery. Late FGR is diagnosed beyond 32 weeks with two solitary parameters: AC or EFW < 3rd centile, or four contributory parameters: EFW or AC < 10th centile, AC or EFW crossing centiles by at least two quartiles on growth charts and cerebroplacental ratio < 5th centile or UA-PI > 95th centile [2].

Fetuses with SGA or FGR are at great risk for perinatal morbidity and mortality, and poor short or long-term neonatal health outcomes, such as impaired neurological and cognitive development, and cardiovascular and endocrine diseases in adulthood [2,3,4,5]. As diagnosis of fetal growth abnormalities is a challenge in everyday practice, several biomarkers were tested if they are sufficient in SGA and FGR prediction, diagnosis, or prognosis of neonatal outcome. Placental dysfunction may be a cause of SGA and FGR and results in al altered expression of placental proteins. Two of these proteins were proved to play a role in preeclampsia (PE) development in singleton gestation: placental growth factor (PlGF) and serum soluble fms-like tyrosine kinase-1 (sFlt-1). In the PROGNOSIS study published in 2016, sFlt-1/PlGF ratio above 38 was proposed as a short-term prediction of the absence of PE in women with a singleton pregnancy in whom the syndrome is clinically suspected [6]. Nowadays PlGF and sFlt-1 are successfully used in common clinical practice [7]. However, data on the usefulness of sFlt-1/PlGF ratio in prediction of adverse perinatal outcome in SGA neonates is still scarce. Considering the above we decided to investigate if sFlt-1/PlGF ratio is related to adverse short-term neonatal outcome in neonates with SGA in women without hypertension or PE and if it could be used in prediction of neonatal complications.

## 2. Materials and Methods

A prospective observational study was conducted at the 2nd Department of Obstetrics and Gynecology, Center of Postgraduate Medical Education, Warsaw, Poland between March, and December 2020. Women in singleton gestation diagnosed with SGA, who gave an inform written consent to participate in the study, were followed from diagnosis to delivery. Gestational age was calculated based on a first day of last menstrual period or a transfer day in assisted reproduction techniques procedures and verified by the crown-rump length measured on the first trimester scan.

Blood samples were collected from all the participants at the time of SGA diagnosis. 10 mL samples of venous blood were collected into polystyrene tubes and mixed with tripotassium versenate (K3-EDTA). PlGF and sFlt-1 serum concentrations were measured using immnunoenzymatic assays. PlGF concentration was analyzed using ELISA Kit for Placenta Growth Factor (Cloud-Clone Corporation, Katy, TX, USA). The lower limit of sensitivity was 15.6 pg/mL. sFlt-1 level was measured with ELISA Kit for Vascular Endothelial Growth Factor Receptor 1 (Cloud-Clone Corporation, Katy, TX, USA) with lower limit of sensitivity at 78 pg/mL. The assays were performed following the manufacturer’s instruction. In all the kits intra-assay precision was <5% and inter-assay precision <10%.

The inclusion criteria contained: age over 18 years old, singleton pregnancy beyond 24 weeks of gestation, ultrasound estimated fetal weight below 10th centile, verified gestational age and complete medical data on the pregnancy outcome. Pregnancies complicated by hypertension, gestational hypertension, PE or lost in the follow up before delivery were excluded from the study. Women were followed weekly on ultrasound and cardiotocography after 26 weeks of gestation. The delivery criteria were: repeated persistent unprovoked fetal heart rate decelerations on cardiotocograph or

- At 26 + 0 to 28 + 6 weeks of gestation: ductus venosus a-wave at or below baseline or fetal heart rate short-term variation (STV) < 2.6 ms;
- At 29 + 0 to 31 + 6 weeks: ductus venosus a-wave at or below baseline or STV < 3.0 ms;
- at 32 + 0 to 33 + 6 weeks: umbilical artery reversed or absent end diastolic flow or STV < 3.5 ms;
- Beyond 34 + 0 weeks: umbilical artery reversed or absent end diastolic flow or STV < 4.5 ms.

SGA was defined as estimated fetal weight <10th centile. PE was diagnosed according to American College of Obstetricians and Gynecologists guidelines [8]. Body mass index (BMI) was calculated by dividing the body mass by the square of the body height. Obesity was defined as BMI 30 and greater. Preterm delivery was diagnosed if a woman gave birth before completed 37 weeks of gestation. sFlt-1/PlGF ratio was calculated by dividing sFlt-1 value by PlGF value.

The primary outcome of the study was neonatal outcome analyzed in the period between birth and discharge home. Apgar score at 5th minute, venous blood pH from umbilical vein, neonatal intensive care unit (NICU) hospitalization, respiratory disorders with use of continuous positive airway pressure (cPAP) or mechanical ventilation, infections treated with intravenous antibiotics, pneumonia, sepsis, circulation failure treated with intravenous catecholamines, necrotizing enterocolitis (NEC), intraventricular hemorrhage grade 1–4 (IVH) and days of hospitalization until discharge home were analyzed. Combined adverse neonatal outcome was considered as at least one of the followings: Apgar score < 7 in the 5th minute, umbilical venous blood pH < 7.0, NICU hospitalization, use of cPAP of mechanical ventilation, pneumonia, sepsis, use of intravenous catecholamines, NEC and IVH grade 3 or 4.

The study protocol was approved by Ethic Committee at the Center for Postgraduate Medical Education and was conducted according to the Declaration of Helsinki.

A power analysis was performed to assess the group sample size. For the 80% power with error probability 0.05 basing on the previously publish results sample size of 76 was adequate to obtain statistical significance of the study results (G*Power version 3.1.9.7). Variables were described as median, interquartile range or percentage. The Mann–Whitney test and the Fisher’s exact test were used for the statistical analysis. *p*-values < 0.05 were considered significant. Cut-off points were estimated based on ROC curves. Sensitivity, specificity, positive and negative predictive values were calculated for the analyzed test. Multivariate logistic regression analysis was performed to adjust for confounding factors and adjusted odds ratios (aOR) were calculated. Data were analyzed using Statistica version 13.1.

## 3. Results

There were 100 women recruited to the study. Eleven were lost in follow up between SGA diagnosis and delivery and therefore were excluded. Seven women developed hypertension or preeclampsia and were excluded from the analysis as well. Finally, there were 82 pregnant women included. Basic characteristics of the study group are presented in Table 1.

All women in the study group were Caucasian. 46 women delivered preterm (56.1%). 29 gave birth before completed 34 weeks (35.4%) and 21 before completed 32 weeks of gestation (25.6%). There were five spontaneous deliveries in the study group (three in the group of women with low and two in the group of women with high ratio). In all other cases labour was induced or elective caesarean delivery was arranged due to abnormal doppler or CTG abnormalities.

The cut-off point for sFlt-1/PlGF ratio was designated on the basis of ROC curve. The cut-off point to predict combined adverse neonatal outcome was 33 with sensitivity of 73% and specificity of 82.2%.

In 44 women sFlt-1/PlGF serum ratio was <33 and in 38 patients ≥ 33. The study group was further divided into two subgroups of women with low (<33) and high (≥33) sFlt-1/PlGF ratio. Characteristics of the two subgroups are presented in Table 1. Women with high ratio gave birth to neonates with lower birthweight at lower gestational age. Serum sFlt-1 concentration was significantly higher, while PlGF significantly lower in women from high ratio group in comparison to low ratio group.

Neonatal outcome in low and high ratio groups is presented in Table 2.

The overall perinatal survival was 79/82. There was one case of intrauterine fetal demise in 26 weeks of gestation in the high ratio group (with sFlt-1/PlGF ratio 311.8). No cases of intrauterine fetal demise were diagnosed in low ratio group. Although there were no differences in umbilical blood pH between the analysed groups, neonates from high ratio group suffered from respiratory disorders and NEC significantly more often. They were hospitalised at NICU more often and were discharged home significantly later. There were no cases of neonatal death in the low ratio group, while two occurred in the high ratio group. One neonate born at 25 weeks of gestation with birthweight of 630 g died on the seventh day of life and the other born at 24 weeks of gestation with birthweight of 320 g strictly after delivery. In both cases spontaneous labour occurred.

sFlt-1/PlGF ratio ≥33 predicted combined neonatal outcome with sensitivity of 73% (95% confidence interval (CI) 56–86.2%), specificity of 82.2% (95% CI 68–92%), positive predictive value of 72% ((%% CI 64.2–84.1%) and negative predictive value of 79% (95% CI 70.2-85.8%). Multivariate logistic regression analysis revealed only two independent risk factors of combined adverse neonatal outcome: neonatal birthweight (aOR 0.98, 95% CI 0.89–0.99) and sFlt-1/plGF ratio (aOR 1.4, 95% CI 1.21–11.89).

## 4. Discussion

We found that sFlt-1/PlGF ratio ≥33 predicted adverse neonatal outcome with high sensitivity and specificity. Women diagnosed with SGA, who had sFlt-1/PlGF ratio ≥33 at the time of SGA diagnosis, gave birth to neonates with lower birthweight and at lower gestational age. Their neonates suffered from respiratory disorders and NEC significantly more often and were hospitalized at NICU more often.

Until now sFlt-1/PlGF ratio was used as a diagnostic tool in SGA or FGR diagnosis in research. Visan et al. conducted a prospective case-control study of women diagnosed with SGA and found ultrasound fetal biometry and maternal risk factors to estimate SGA with the sensitivity of 44.4% and a specificity of 89% for a false positive result of 10%. After adding sFlt1/PIGF ratio to the ultrasound fetal biometry and maternal risk factors, the sensitivity increased to 84.21% with the specificity of 84.31% for a false positive rate of 10%. The authors concluded that when associated with maternal factors and ultrasound biometry, the sFlt1/PIGF ratio enhanced the sensitivity for detecting SGA [9]. Efficacy of sFlt-1/PlGF ratio in FGR prediction was assessed by other researchers as well. Herraiz et al. reported results from the prospective cohort study. They followed 5601 singleton pregnancies from measurement of the sFlt-1/PlGF ratio at 24–28 weeks of gestation until delivery. sFlt-1/PlGF ratio >95th centile showed a sensitivity of 100% (95% CI 78.5–100) and specificity of 80.6% (95% CI 75.0–85.2) in prediction of PE and/or FGR. The test performance was optimal to predict PE/FGR requiring delivery before 32 weeks [10]. Hendrix et al. performed a systematic-review to explore the predictive performance of maternal concentrations of PlGF, sFlt-1 and their ratio for FGR and SGA at different gestational ages. According to the authors the biomarkers can be a valuable screening tool for SGA pregnancies, but unfortunately, there is not yet a clear cut-off value to use for screening. More research is needed to see if these biomarkers are sufficiently able to differentiate growth restriction on their own and how these biomarkers in combination with other relevant clinical and ultrasound parameters can be used in clinical routine diagnostics [11].

Relation between increased sFlt-1/PlGf ratio and adverse neonatal outcome was reported by several authors [12,13,14,15]. Garcia-Manau et al. observed sFlt-1/PlGF values and pregnancy outcomes among early-onset SGA/FGR. The authors found significant correlation between greater sFlt-1/PlGF ratio values and gestational age at delivery, time from diagnosis to delivery, birthweight z-scores, and time in NICU (r = −0.637, r = −0.576, r = −0.161, and r = 0.311, respectively). Therefore, they concluded that sFlt-1/PlGF could aid in early-onset FGR/SGA severity classification and clinical management when Doppler assessment is not feasible [16]. In our study sFlt-1/PlGF ratio was significantly related to birthweight and NICU hospitalization as well. Chang et al. measured sFlt-1 and PlGF in women with SGA or PE during gestational age of 16–36 weeks in 25 pregnant women. In their group of 13 women with sFlt-1/PlGF >85 two had intrauterine fetal demise and one underwent artificial termination of pregnancy due to poor fetal outcome. The surviving offspring in this group had higher incidences of preterm birth, lower birth weight, respiratory distress syndrome, and bronchopulmonary dysplasia [17]. In our study we found a significantly higher incidence of respiratory disorders in neonates from high sFlt-1/PlGF ratio group as well. Another prospective observational study of singleton pregnancies with SGA between 20 + 0 and 31 + 6 weeks of gestational age was conducted by Mendoza et al. Adverse perinatal outcomes occurred in 81 (55.9%) pregnancies. The authors developed an individual risk assessment model, which included sFlt-1/PlGF ratio measurement. The model can be used at the time of early-onset SGA diagnosis and permits accurate counseling of parents with an affected fetus [18].

Shim et al. measured sFlt-1/PlGF ratio in 47 pregnant women with SGA and 461 controls with appropriate for gestational age fetuses. The mean sFlt-1/PlGF ratio at 24 to 28 + 6 weeks of gestation was significantly higher in SGA with averse neonatal outcome group than in the control group (14.42 ± 23.8 vs. 109.12 3.96, *p* = 0.04) and the ratio retained an independent and significant association with SGA with adverse neonatal outcomes (odds ratio = 1.017, *p* = 0.01). sFlt-1/PlGF ratio cut-off of 28.15 at 29 to 36 + 6 weeks significantly predicted adverse outcomes among SGA neonates (sensitivity = 76.9%, specificity = 88%) [19]. Our cut-off of 33 predicted combined adverse neonatal outcome with similar sensitivity of 73% and specificity of 82.2%. However, our study group was almost twice as large as in the study by Shim et al.

As sFlt-1/PlGF was proved to be effective in prediction of adverse neonatal outcome in SGA, there is a need to develop risk assessment models and clinical guidelines. In multicenter, placebo-controlled STRIDER UK randomized trial of singleton pregnancies with severe early-onset fetal growth restriction a prediction model for short-term neonatal outcomes was developed. It contained maternal demographics (age, parity, blood pressure, PE, gestational hypertension), fetal biometric (estimated fetal weight) and Doppler measurements (middle cerebral artery, umbilical artery) and maternal angiogenic biomarkers (PlGF, soluble endoglin (sEng), sFlt-1 and sFlt-1/PlGF ratio). Multivariate regression analysis identified sFlt-1/PlGF as independent predictor of livebirth (sFlt-1/PlGF ratio odds ratio (OR): 0.53 (95% CI 0.284–0.994) and overall survival (sFlt-1/PlGF ratio OR 0.51 (95% CI 0.286–0.904) [20].

The strength of our study is its homogenous study group including only normotensive women diagnosed with SGA. The sample size was assessed using power analysis. Inclusion criteria and outcome are well defined. However, there are some limitations. It is a single center study with a short time of neonatal observation period.

## 5. Conclusions

sFlt-1/PlGF ratio is a useful toll in prediction of short-term adverse neonatal outcome in SGA pregnancies. Further large multicenter prospective studies with long time follow up are required to investigate correlation between maternal sFlt-1/PlGF ratio and long-term neonatal outcome in order to develop a clinically useful model for prediction of adverse neonatal outcome in SGA neonates.

## Figures and Tables

**Table 1:** Basic characteristics of the study group.

	Study Group N = 82	sFlt-1/PlGF Ratio <33 *n* = 44	sFlt-1/PlGF Ratio ≥33 *n* = 38	*p*
	number (%)			
age (years) *	30 (26.8–33.4)	31 (27.6–34)	28 (26.3–31.6)	0.06
primiparity	43 (52.4)	20 (45.5)	23 (60.5)	0.2
PE in previous pregnancy	6 (7.3)	2 (4.5)	4 (10.5)	0.4
SGA in previous pregnancy	9 (11)	5 (11.4)	4 (10.5)	0.9
preterm delivery in previous pregnancy	5 (6.1)	3 (6.8)	2 (5.3)	1
BMI (kg/m^2^) *	23.4 (20.8–29.3)	23.2 (21.7–28.9)	23.7 (20.6–30)	0.8
obesity	3 (3.7)	2 (4.5)	1 (2.6)	0.8
gestational age at SGA diagnosis (weeks) *	32 (28.4–33)	32 (28.5–34)	31 (27–32)	0.03
FGR	41 (50)	13 (29.5)	28 (73.7)	<0.001
sFlt-1 (pg/mL) *	2462.5 (1378–9720)	1429.9 (1127–2310)	7151.5 (3890–1498)	<0.001
PlGF (pg/mL) *	136 (49–598)	323.2 (172–698)	50 (33.5–63)	<0.001
sFlt-1/PlGF ratio	15.5 (3–280)	4.6 (2.6–9)	198 (69–359)	<0.001
gestational age at delivery (weeks) *	36 (33–37)	37 (34–37)	31 (27–37)	<0.001
neonate birthweight (g) *	2080 (1780–2450)	2420 (2100–2660)	1410 (835–1960)	<0.001
birthweight centile *	6 (0–13)	7 (2–13)	<1 (0–3)	<0.001
corticosteroids	44 (53.7)	16 (36.4)	28 (73.7)	0.003
magnesium sulfate	17 (20.7)	2 (4.5)	15 (39.5)	<0.001
caesarean delivery	52 (63.4)	16 (36.4)	36 (94.7)	<0.001
labour induction	42 (51.2)	38 (86.4)	2 (5.2)	<0.001

*—median (interquartile range) PE—preeclampsia; SGA—small for gestational age; FGR—fetal growth restriction; BMI—body mass index; sFlt-1—soluble fms-like tyrosine kinase-1; PlGF—placental growth factor.

**Table 2:** Neonatal outcome in sFlt-1/PlGF ratio <33 and ≥33 groups.

	sFlt-1/PlGF Ratio <33 *n* = 44	sFlt-1/PlGF Ratio ≥33 *n* = 38	*p*
	number (%)		
5 min Apgar score *	10 (10–10)	10 (8–10)	0.9
umbilical vein blood pH *	7.36 (7.31–7.4)	7.36 (7.31–7.38)	0.8
NICU	7 (15.9)	25 (67.8)	<0.001
cPAP	5 (11.4)	26 (68.4)	<0.001
mechanical ventilation	2 (4.5)	12 (31.6)	0.002
antibiotics	6 (13)	14 (38.9)	0.02
sepsis	3 (6.8)	4 (10.5)	0.5
NEC	0	4 (10.5)	0.004
IVH 1/2	1 (2.3)	4 (10.5)	0.1
IVH 3/4	0	0	1
circulation failure	1 (2.3)	5 (13.2)	0.09
pneumonia	1 (2.3)	3 (7.9)	0.3
days of hospitalisation *	5 (3–9)	27 (10–48)	<0.001

*—median (interquartile range); NICU—neonatal intensive care unit; NEC—necrotizing enterocolitis; IVH 1/2—intraventricular hemorrhage grade 1 or 2; IVH 3/4—intraventricular hemorrhage grade 3 or 4.

## Data Availability

Data is available on request from the corresponding author.
